# Comparison of plasma mitochondrial DNA copy number in asymptomatic and symptomatic COVID-19 patients

**DOI:** 10.3389/fmicb.2023.1256042

**Published:** 2023-10-06

**Authors:** Shahrzad Shoraka, Seyed Reza Mohebbi, Seyed Masoud Hosseini, Mohammad Reza Zali

**Affiliations:** ^1^Department of Microbiology and Microbial Biotechnology, Faculty of Life Sciences and Biotechnology, Shahid Beheshti University, Tehran, Iran; ^2^Gastroenterology and Liver Diseases Research Center, Research Institute for Gastroenterology and Liver Diseases, Shahid Beheshti University of Medical Sciences, Tehran, Iran

**Keywords:** mitochondrial DNA, cell-free nucleic acids, SARS-CoV-2, COVID-19, biomarkers

## Abstract

**Introduction:**

Since the beginning of the COVID-19 pandemic, a wide clinical spectrum, from asymptomatic infection to mild or severe disease and death, have been reported in COVID-19 patients. Studies have suggested several possible factors, which may affect the clinical outcome of COVID-19. A pro-inflammatory state and impaired antiviral response have been suggested as major contributing factors in severe COVID-19. Considering that mitochondria have an important role in regulating the immune responses to pathogens, pro-inflammatory signaling, and cell death, it has received much attention in SARS-CoV-2 infection. Recent studies have demonstrated that high levels of cell-free mitochondrial DNA (*cf*-mtDNA) are associated with an increased risk of COVID-19 intensive care unit (ICU) admission and mortality. However, there have been few studies on *cf*-mtDNA in SARS-CoV-2 infection, mainly focusing on critically ill COVID-19 cases. In the present study, we investigated *cf*-mtDNA copy number in COVID-19 patients and compared between asymptomatic and symptomatic cases, and assessed the clinical values. We also determined the *cf*-nuclear DNA (*cf*-nDNA) copy number and mitochondrial transcription factor A (TFAM) mRNA level in the studied groups.

**Materials and methods:**

Plasma and buffy coat samples were collected from 37 COVID-19 patients and 33 controls. Briefly, after total DNA extraction, plasma *cf*-mtDNA, and *cf*-nDNA copy numbers were measured by absolute qPCR using a standard curve method. Furthermore, after total RNA extraction from buffy coat and cDNA synthesis, TFAM mRNA levels were evaluated by qPCR.

**Results:**

The results showed that *cf*-mtDNA levels in asymptomatic COVID-19 patients were statistically significantly higher than in symptomatic cases (*p* value = 0.01). However, *cf*-nDNA levels were higher in symptomatic patients than in asymptomatic cases (*p* value = 0.00). There was no significant difference between TFAM levels in the buffy coat of these two groups (*p* value > 0.05). Also, *cf*-mtDNA levels showed good diagnostic potential in COVID-19 subgroups.

**Conclusion:**

*cf*-mtDNA is probably important in the outcome of SARS-CoV-2 infection due to its role in inflammation and immune response. It can also be a promising candidate biomarker for the diagnosis of COVID-19 subgroups. Further investigation will help understanding the COVID-19 pathophysiology and effective diagnostic and therapeutic strategies.

## 1. Introduction

Although the majority of COVID-19 patients can develop an adequate immune response that eventually leads to viral clearance and recovery, a significant number of patients present with severe clinical manifestations that require intensive care treatment (Bohn et al., 2020). Since the beginning of the recent pandemic, many factors have been suggested that may affect the clinical outcome of COVID-19, such as host-related factors including age, comorbidities, and immunity status (Zhang et al., 2020; Adab et al., 2022). Severe COVID-19 seems to be associated with a dysregulated immune response and cytokine storm-mediated inflammation (Elbadawy et al., 2023). Since mitochondria play an important role in the immune and pro-inflammatory responses, recent studies are paying increasing attention to this organelle in the COVID-19 pathogenesis (Valdés-Aguayo et al., 2021; Shoraka et al., 2023).

Mitochondria is an organelle in eukaryotic cells that have important roles in biological processes including physiological and pathological events (Riley and Tait, 2020). Mitochondria also play an essential role in host antiviral signaling via mitochondrial antiviral-signaling (MAVS) protein (Costa et al., 2022; Faizan et al., 2022). Following virus entry into the host cell, viral RNA is recognized by pattern recognition receptors (PRRs) (such as RIG-I and MDA5 sensors) and activate immune responses dependent on MAVS, a protein located in the outer membrane of mitochondria. MAVS acts as a platform for downstream signaling and could mediate the activation of NF-κB and interferon regulatory factor 3 and 7 (IRF3/7). The NF-κB signaling pathway subsequently promotes the expression of several pro-inflammatory cytokines. The IRF3/7 pathway induces antiviral genes, such as type I interferon (IFN-I) and IFN-stimulated genes (ISGs), which prevent viral replication and transmission (Ren et al., 2020).

Furthermore, mitochondrial components including mitochondrial DNA (mtDNA) have been described as damage-associated molecular patterns (DAMPs) which, like pathogen-associated molecular patterns (PAMPs), are recognized by PRRs, and then lead to activation of inflammatory signaling pathways (De Gaetano et al., 2021). The release of mtDNA fragments into the extracellular space, such as blood, could trigger systemic inflammation (Mahmoodpoor et al., 2022). The inflammatory role of mitochondrial products, especially mtDNA, is indirectly attributed to mitochondrial endosymbiosis. Since mitochondria have genetic homology with prokaryotes, recognition of the mitochondrial genome in the extracellular space by the immune system leads to the initiation of inflammatory responses (Ali et al., 2020; De Gaetano et al., 2021). mtDNA is also a crucial mediator of the antiviral response that promotes inflammation through innate immune pathways, such as toll-like receptor 9 (TLR9), cGAS-STING, and the inflammasome (Hepokoski et al., 2022). In previous years, studies have emphasized the role of mitochondrial DNA in inflammation and immunity (Riley and Tait, 2020; Newman and Shadel, 2023).

The mtDNA is a 16.5 kb circular double-stranded DNA that contains 37 genes and is present at a high copy number per cell (Filograna et al., 2021). The mtDNA copy number is mainly regulated by several nuclear-encoded transcription and replication factors, including transcription factor A (TFAM) (Sourty et al., 2022). Many studies have shown the presence of mtDNA fragments in the cytosol, outside of the cell or in the circulation following mitochondrial damage (Heilig et al., 2023; Newman and Shadel, 2023; Xu et al., 2023).

In recent years, studies have increasingly pointed to the association between circulating mitochondrial DNA (*cf*-mtDNA) level and pathological conditions such as inflammation, trauma, and viral infections (Mohamed et al., 2016; Arshad et al., 2018; Ali et al., 2020; Faust et al., 2020; Kim et al., 2021). Increased *cf*-mtDNA level is closely related to disease progression (Cossarizza et al., 2011; Faust et al., 2020). In addition to mtDNA being a key mediator of immune responses, *cf*-mtDNA may serve as a potential clinical biomarker (De Gaetano et al., 2021; Hepokoski et al., 2022).

Although little is known about the impact of SARS-CoV-2 on mitochondria, there is evidence of morphological and functional mitochondrial alterations during SARS-CoV-2 infection. In addition to impaired antiviral signaling and mitochondria damage, SARS-CoV-2 infection is also associated with high levels of extracellular mtDNA (Cortese et al., 2020; Archer et al., 2022; Dodig et al., 2022; Faizan et al., 2022). Recently, a 2021 study showed that elevated circulating mtDNA levels may be correlated with COVID-19 poor outcomes (Scozzi et al., 2021). Some other studies suggested *cf*-mtDNA as a potential biomarker for predicting mortality in COVID-19 (Edinger et al., 2022; Hepokoski et al., 2022). However, all these studies evaluated mtDNA in critically ill hospitalized COVID-19 cases and did not compare *cf*-mtDNA levels among COVID-19 patients with different disease severity.

Considering that the pathophysiological mechanisms in the progression of COVID-19 are unclear, further research is warranted (Bohn et al., 2020). In the present study, cell-free mtDNA copy number was evaluated in patients with COVID-19, compared between asymptomatic and symptomatic patients, and clinical values were investigated. The *cf*-nuclear DNA copy number and TFAM mRNA levels in the studied groups were also determined.

## 2. Materials and methods

### 2.1. Study design

The present study was performed with the approval of the ethics committee of the Research Institute for Gastroenterology and Liver Disease (RIGLD) in Tehran, Iran (#IR.SBMU.RIGLD.1399.008) and informed consent was obtained from all participants before being included in the study. We recruited 37 COVID-19 patients who referred to Taleghani and Imam Hossein Educational Hospitals, Shahid Beheshti University of Medical Sciences (Tehran, Iran), and also 33 healthy controls. Clinical data were extracted from the patients and medical records.

The selection criteria for the COVID-19 group were based on clinical features, chest computed tomography (CT) scan images, and positive SARS-CoV-2 RT-qPCR test on nasopharyngeal swabs. The exclusion criteria were: (Bohn et al., 2020) patients without RT-qPCR test results, (Zhang et al., 2020) those who were not laboratory confirmed (RT-qPCR cycling threshold (Ct) >40) despite clinical and radiological presentation of COVID-19, (Adab et al., 2022) who had previously COVID-19 vaccination, (Elbadawy et al., 2023) who had history of COVID-19 infection (confirmed by qPCR or serological test), (Valdés-Aguayo et al., 2021) COVID-19 cases with bacterial and/or viral co-infection. The inclusion criteria for healthy controls were: (Bohn et al., 2020) individuals must have a negative RT-qPCR test for SARS-CoV-2 and, (Zhang et al., 2020) must have no history of COVID-19 (confirmed by qPCR or serological test) and, (Adab et al., 2022) must not have received the COVID-19 vaccine and, (Elbadawy et al., 2023) without any comorbidities.

### 2.2. Classification of COVID-19 patients according to the disease severity

On the day of hospital admission, a standard questionnaire containing questions about medical history, medication use, and lifestyle was answered by the patients. To classify the disease severity, the following clinical variables were also measured: body temperature, heart and respiration rate, blood pressure, and oxygen saturation. Laboratory data including hemoglobin, blood urea nitrogen (BUN), albumin, serum electrolytes, glucose, coagulation tests, creatine kinase (CK), and C-reactive protein (CRP) were also evaluated. After analyzing the clinical and laboratory parameters, the COVID-19 patients were classified as mild/moderate and severe.

COVID-19 patients with any of the following characteristics were classified into the severe group: (Bohn et al., 2020) dyspnea (≥30 breath/min) and/or, (Zhang et al., 2020) low blood O_2_ saturation without oxygen supply (≤ 93%) and/or, (Adab et al., 2022) PaO2/FiO2 index (< 300) and/or, lung infiltration on thorax CT scan (>50%). Laboratory-confirmed COVID-19 patients without any clinical symptoms were classified into asymptomatic cases. And other COVID-19 patients with symptoms such as fever, muscle pain, cough, and sore throat and findings of mild to moderate pneumonia on lung CT scan were categorized as mild/moderate group (Kocak et al., 2021).

According to these criteria, the COVID-19 patients were divided into the following subgroups: asymptomatic (17 patients), mild/moderate (12 patients), and severe (8 patients).

### 2.3. Sample collection and preparation

Blood sampling from symptomatic COVID-19 patients (20 patients in total) was collected on three separate days (days 1, 3, and 5 after enrollment), while those of asymptomatic patients and controls were collected once. Venous blood samples were obtained in EDTA containing tube. Plasma and buffy coats were separated by centrifugation (Eppendorf 5810R). Aliquots were stored frozen at −80°C.

### 2.4. Total cell-free DNA extraction from plasma

Total cell-free DNA (t-cfDNA) was extracted from 200 μL plasma samples using the QIAamp® DNA Mini Kit (Qiagen, Cat. No. 51304) as described by the manufacturer. The quantity and quality of extracted DNA were carried out by Nanodrop (Thermo Scientific). The purified DNA samples were stored at −20°C for future testing.

### 2.5. Plasmid and standard curve preparation

To convert the results to copy number per microliter (μL), a standard curve generated using a plasmid containing human mtDNA and a plasmid containing human nuclear DNA (nDNA) was used. Selected regions of purified mitochondrial DNA (NADH dehydrogenase 1 (ND1)) and nuclear DNA (β-globin hemoglobin (HBB)) were amplified by conventional PCR using the following primers, respectively:

5′- CCACCTCTAGCCTAGCCGTTTA-3′ (forward) and

5′- GGGTCATGATGGCAGGAGTAAT-3′ (reverse) for ND1 (mitochondrial DNA),

5′- ACTGGAGTAAAGGAAATGGAC-3′ (forward) and

5′- TTGCTTCTACTCTGTGAATGG-3′ (reverse) for HBB (nuclear DNA).

Each PCR product was inserted into a pGEM®-T Easy Vector System I (Promega, Cat. No. A1360) following the manufacturer’s protocol. Competent *E. coli* DH5α was chemically transformed with each recombinant plasmid, separately. After the bacterial transformation, the plasmid DNA was extracted from selected colonies using by QIAprep® Spin Miniprep kit (Qiagen, Cat. No. 27104).

To prepare appropriate serial dilutions to obtain a standard curve, the plasmid copy number was quantified. The copy number for each plasmid was estimated by the following formula:


$$\frac{DNA\;\mathrm{concentration}\;\left(\mathrm{ng}/ul\right)\times {\mathrm{Avogadro}}^{\prime }s\;\mathrm{number}}{\mathrm{Length}\;\mathrm{of}\;\mathrm{template}\;\left(\mathrm{bp}\right)\times \mathrm{Conversion}\;\mathrm{factor}\;to\;\mathrm{ng}\times \mathrm{Average}\;\mathrm{weight}\;\mathrm{of}\;a\;\mathrm{base}\;\mathrm{pair}\;\left(\mathrm{Da}\right)}$$

Avogadro’s number = 6.022 × 10^23^, Conversion number to nanogram (ng) = 10^9^, Average weight of a base pair (Da) = 650

A serial dilution of each stock solution was prepared to generate a standard curve (range: 10–10^9^ copies/μL).

### 2.6. Measurement of *cf*-mtDNA and *cf*-nDNA copy numbers by qPCR

The mtDNA and nDNA copy numbers were measured by absolute quantification using RealQ plus 2x Master Mix Green (Ampliqon, Cat. No. A324406) and Rotor-Gene Q real-time PCR cycler (Qiagen). The mitochondrial ND1 gene and the nuclear HBB gene were used to quantify mtDNA and nDNA copy numbers, respectively. The primer sequences previously described.

The qPCR thermal conditions were 95°C for 15 min, 35 cycles of denaturation at 95°C for 15 s, and annealing at 60°C for 60 s. A melting curve analysis was added to verify the specificity of the PCR products.

To obtain absolute quantification, two standard curves were plotted with a 5-point standard dilution of known copy numbers of mtDNA-containing and nDNA-containing plasmids in each run separately. A negative no-template control also was included in each set of the run. All samples were run in duplicate to ensure accuracy. The cycle threshold (Ct) variation was less than one cycle between duplicates within the same run.

### 2.7. Total RNA extraction from buffy coat

Total RNA was isolated from the buffy coat using RiboEx™ reagent (GeneAll, Cat. No. 301-001) as described by the manufacturer. The quality of extracted RNA was evaluated by Nanodrop (Thermo Scientific). For cDNA synthesis, total RNA was reversed transcribed using AddScript cDNA synthesis kit (Addbio, Cat. No.22701). cDNA samples were stored at −20°C until real-time PCR analysis.

### 2.8. Evaluation of TFAM mRNA levels by qPCR

The qPCR was performed by Rotor-Gene Q real-time PCR cycler (Qiagen) and RealQ plus 2x Master Mix Green (Ampliqon, Cat. No. A324406) according to the manufacturer’s provided instructions. The primer sequences were:

5′- GGCAAGTTGTCCAAAGAAACC -3′ (forward) and

5′- GCATCTGGGTTCTGAGCTTTA −3′ (reverse) for TFAM,

5’-TGCTGTCTCCATGTTTGATGTATCT-3′ (forward) and

5’-TCTCTGCTCCCCACCTCTAAGT-3′ (reverse) for β2-Microglobulin as an internal control.

The qPCR cycling was run as follows: 95°C for 15 min, 35 cycles of denaturation at 95°C for 15 s, and annealing at 60°C for 60 s. At the end of the reaction, a melt curve analysis was performed to confirm the absence of non-specific amplified products. All samples were run in duplicate. The 2^-ΔCt^ method was used to determine the expression level of TFAM relative to internal control β2-Microglobulin.

### 2.9. Statistical analysis

All the statistical analyses were calculated using SPSS version 22 software and all figures were prepared using GraphPad Prism version 8.0.1. A *p* value < 0.05 defined as statistically significant. Continuous variables were represented as the means ± standard deviation (SD) represented for normal numeric distribution. The median (interquartile range [IQR]) was presented for non-normal distribution. Variables with normal distribution were analyzed by parametric tests (t-test and ANOVA), and variables with non-normal distribution were compared using non-parametric tests (Mann–Whitney *U* test and Kruskal-Wallis test). Also, the *χ*^2^ test was applied to compare discrete variables. The Spearman correlation analysis was performed to determine variable correlations. Receiver-operating characteristic (ROC) curve analysis and area under the curve (AUC) were performed to determine the feasibility of using each variable as a biomarker. The AUC values were classified as excellent (>0.90), good (0.80–0.89), adequate (0.70–0.79), poor (0.51–0.69), and insufficient (<0.50). The cut-off values were selected to maximize Yuden’s index.

## 3. Results

### 3.1. Demographics and clinical characteristics of the participants

According to inclusion and exclusion criteria, blood samples were collected from 33 healthy controls and 37 COVID-19 patients. To avoid confounding effects, we matched cases and controls by gender and age (±5 years). Basic characteristics of studied groups ate shown in Table 1. There was a significant difference between the mean age of asymptomatic (42.94 ± 10.29 years) and symptomatic (58.60 ± 13.72 years) COVID-19 patients (*p* value < 0.05), and also mild/moderate (53.00 ± 13.73) and severe (67.00 ± 8.97) subgroups (*p* value < 0.05). Therefore, the analyses were adjusted for age.

**Table 1:** Demographic data of studied groups.

	Healthy controls	COVID-19 patients (asymptomatic and symptomatic)	*p* value
Number of subjects	33	37	
Age			
Mean ± SD	47.87 ± 14.69	51.40 ± 14.45	0.31
Range (years)	25–78	32–79	
Gender			
Male, n (%)	20 (60)	25 (67)	0.54
Female, n (%)	13 (40)	12 (33)	
COVID-19 patients			
	Asymptomatic	Symptomatic	
Number of subjects	17	20	
Age			
Mean ± SD	42.94 ± 10.29	58.60 ± 13.72	**0.00**
Range (years)	33–65	32–79	
Gender			
Male, n (%)	12 (70)	13 (65)	0.71
Female, n (%)	5 (30)	7 (35)	
Symptomatic COVID-19 patients			
	Mild/moderate	Severe	
Number of subjects	12	8	
Age			
Mean ± SD	53.00 ± 13.73	67.00 ± 8.97	**0.02**
Range (years)	32–78	50–79	
Gender			
Male, n (%)	9 (75)	4 (50)	0.25
Female, n (%)	3 (25)	4 (50)	

Significant *p* values are highlighted.

Also, detailed clinical characteristics and laboratory parameters of COVID-19 patients groups are shown in Tables 2, 3, respectively. As shown in Table 2, there is a significant difference in the rate of hospitalization and intensive care unit (ICU) admission in mild/moderate and severe COVID-19 groups (*p* values < 0.05). All hospitalized COVID-19 patients recovered and no deaths occurred. Also, underlying diseases were significantly more in the symptomatic COVID-19 patients than in the asymptomatic group. There was a statistically significant difference between asymptomatic and severe groups in terms of underlying disease (*p* values < 0.05), while there was no significant difference between the mild/moderate and severe COVID-19 groups (*p* value > 0.05). As shown in Table 3, many laboratory parameters in symptomatic COVID-19 cases were outside the normal range. The O_2_ saturation, white blood cell, neutrophil percentage, total bilirubin, direct bilirubin, creatine kinase-MB, D-Dimer, and CRP are significantly different between the mild/moderate and severe subgroups (*p* values < 0.05). No additional significant difference was found in general clinical features and laboratory parameters between mild/moderate and severe COVID-19 patients (*p* values > 0.05).

**Table 2:** Clinical features of COVID-19 patients.

Clinical features	Symptomatic COVID-19 patients (*n* = 20)			*p* value
	Mild/moderate (*n* = 12)	Severe (*n* = 8)		
General				
Fever, n (%)	6 (50)	2 (25)		0.26
Myalgia, n (%)	4 (33)	3 (37)		0.84
Headache, n (%)	2 (16)	0		0.34
Tachycardia, n (%)	1 (8)	0		0.40
Pulmonary				
Dry cough, n (%)	7 (58)	4 (50)		0.71
Chest pain, n (%)	1 (8)	3 (37)		0.11
Dyspnea, n (%)	9 (75)	3 (37)		0.09
ARDS, n (%)	0	2 (25)		0.06
Gastrointestinal				
Diarrhea, n (%)	2 (16)	3 (37)		0.29
Nausea, n (%)	3 (25)	1 (12)		0.49
Anorexia, n (%)	2 (16)	2 (25)		0.64
In-hospital care				
Hospitalization, n (%)	7 (58)	8 (100)		**0.03**
Intensive care unit, n (%)	0	3 (37)		**0.02**
Endotracheal intubation, n (%)	0	2 (25)		0.68
In-hospital mortality, n (%)	0	0		-
	Asymptomatic (*n* = 17)	Symptomatic (*n* = 20)		
		Mild/Moderate (*n* = 12)	Severe (*n* = 8)	
Underlying disease				
Diabetes mellitus, n (%)	0	2 (16)	1 (12)	0.23
Chronic kidney disease, n (%)	0	0	1 (12)	0.15
Cardiac, n (%)	1 (5)	1 (8)	0	0.71
Cancer, n (%)	0	1 (8)	2 (25)	0.10
Hypertension, n (%)	1 (5)	1 (8)	2 (25)	0.33
Total, n (%)	2 (11)	5 (41)	6 (75)	**0.00**

Significant *p* values are highlighted.

**Table 3:** Laboratory results of symptomatic COVID-19 patients.

Laboratory values	Normal range (In adults)	COVID-19 patients		*p* value
		Mild/moderate (*n* = 12)	Severe (*n* = 8)	
Vital sign				
Systolic blood pressure (mmHg), mean ± SD	<120	125.4 ± 15.88	125.6 ± 31.10	0.98
Diastolic blood pressure (mmHg), mean ± SD	<80	79.25 ± 13.51	81.33 ± 16.08	0.77
Heart rate (bpm), mean ± SD	60–100	80.90 ± 14.20	82.00 ± 8.86	0.85
Respiratory rate (brpm), mean ± SD	12–16	17.44 ± 1.42	17.17 ± 1.60	0.73
O_2_ saturation (%), mean ± SD	95–100	93.36 ± 1.80	86.86 ± 5.90	**0.00**
Temperature (°C), mean ± SD	36.5–37.3	37.01 ± 1.16	36.93 ± 0.46	0.86
Blood count				
Red blood cell (×10^6^/μL), mean ± SD	3.2–4.6	3.79 ± 0.52	4.24 ± 0.57	0.33
White blood cell (×10^3^/μL), mean ± SD	4–11	8.72 ± 5.26	14.38 ± 7.38	**0.04**
Lymphocytes (%), mean ± SD	20–40	22.77 ± 16.90	16.50 ± 10.86	0.36
Neutrophils (%), mean ± SD	40–60	67.50 ± 6.45	80.60 ± 7.79	**0.03**
Platelet (×10^3^/μL), mean ± SD	150–400	233.10 ± 79.70	226.10 ± 131.60	0.88
Hemoglobin (g/dL), mean ± SD	12–15*	11.96 ± 2.26	10.24 ± 3.17	0.19
Biochemistry parameters				
SGPT (IU/L), mean ± SD	<31	38.80 ± 38.76	40.55 ± 30.05	0.91
SGOT (IU/L), mean ± SD	<38	43.33 ± 31.38	37.31 ± 38.08	0.71
Alkaline phosphatase (U/L), mean ± SD	64–306	238.80 ± 59.46	226.80 ± 42.54	0.73
Direct bilirubin (mg/dL), mean ± SD	<0.3	0.30 ± 0.14	11.55 ± 2.05	**0.01**
Total bilirubin (mg/dL), mean ± SD	0.1–1.2	0.65 ± 0.07	17.35 ± 7.99	**0.05**
Albumin (g/dL), mean ± SD	3.5–5	4.05 ± 0.28	3.90 ± 1.15	0.80
Creatine kinase-MB (U/L), mean ± SD	<24	15.00 ± 7.07	32.00 ± 16.52	**0.03**
Sodium (meq/L), mean ± SD	135–145	139.70 ± 3.75	140.40 ± 10.36	0.82
Potassium (meq/L), mean ± SD	3.5–5	4.00 ± 0.33	4.20 ± 0.58	0.34
Creatinine (mg/dL), mean ± SD	0.6–1.3*	1.00 ± 0.31	2.41 ± 3.24	0.16
Blood urea nitrogen (BUN) (mg/dL), mean ± SD	5–20	28.25 ± 26.83	26.83 ± 15.43	0.91
Blood sugar (mg/dL), mean ± SD	170–200	131.30 ± 50.65	112.50 ± 37.99	0.59
Coagulation marker				
Partial Thromboplastin Time (PTT) (sec), mean ± SD	24–42	34.75 ± 2.21	36.50 ± 2.38	0.32
INR, mean ± SD	<1.1	1.05 ± 0.05	1.52 ± 0.65	0.19
Inflammation marker				
Lactate Dehydrogenase (LDH) (IU/L), mean ± SD	230–460	461.40 ± 205.10	473.00 ± 86.01	0.90
ESR (mm/h), mean ± SD	<30	30.55 ± 26.30	44.71 ± 34.52	0.33
D-Dimer (ng/mL), mean ± SD	<600	603.00 ± 778.50	157.40 ± 593.90	**0.04**
C-reactive protein (CRP) (mg/L), mean ± SD	<6	17.86 ± 23.60	65.00 ± 38.66	**0.00**
SARS-CoV-2 serology				
Positive IgM nucleocapsid, n (%)		9 (75)	2 (25)	0.28
Positive IgG nucleocapsid, n (%)		7 (58)	3 (37)	0.36

*The normal values are gender-dependent. Significant *p* values are highlighted.

Also, laboratory parameters for symptomatic COVID-19 patients on days 1, 3 and 5 are shown in Table 4. No significant differences in clinical parameters were observed between these days.

**Table 4:** Laboratory data for follow-up COVID-19 patients.

Laboratory values	Normal range (In adults)	COVID-19 patients			*p* value
		Day 1	Day 3	Day 5	
Blood count					
White blood cell (×10^3^/μL), mean ± SD	4–11	10.99 ± 6.64	8.10 ± 5.11	10.38 ± 11.33	0.61
Lymphocytes (%), mean ± SD	20–40	20.26 ± 14.79	19.06 ± 12.32	20.03 ± 11.49	0.98
Neutrophils (%), mean ± SD	40–60	74.13 ± 10.13	76.24 ± 13.38	73.30 ± 16.73	0.91
Platelet (×10^3^/μL), mean ± SD	150–400	230.30 ± 100.40	183.80 ± 69.98	192.70 ± 67.77	0.25
Hemoglobin (g/dL), mean ± SD	12–15*	10.08 ± 4.32	10.24 ± 2.47	10.11 ± 2.05	0.99
Biochemistry parameters					
SGPT (IU/L), mean ± SD	<31	50.51 ± 52.93	75.33 ± 78.11	59.17 ± 41.88	0.64
SGOT (IU/L), mean ± SD	<38	37.49 ± 34.43	34.33 ± 25.99	46.33 ± 25.90	0.78
Sodium (meq/L), mean ± SD	135–145	140.00 ± 6.91	138.40 ± 4.47	139.00 ± 4.04	0.78
Potassium (meq/L), mean ± SD	3.5–5	4.08 ± 0.45	4.01 ± 0.60	3.96 ± 0.41	0.78
Creatinine (mg/dL), mean ± SD	0.6–1.3*	1.52 ± 2.13	2.12 ± 2.77	2.24 ± 3.43	0.70
Blood urea nitrogen (BUN) (mg/dL), mean ± SD	5–20	27.64 ± 21.90	39.88 ± 27.21	32.59 ± 32.90	0.53
Coagulation marker					
INR, mean ± SD	<1.1	1.28 ± 0.49	1.57 ± 0.81	1.46 ± 0.57	0.71
Inflammation marker					
Lactate Dehydrogenase (LDH) (IU/L), mean ± SD	230–460	539.20 ± 240.30	351.50 ± 89.80	362.30 ± 236.70	0.35
C-reactive protein (CRP) (mg/L), mean ± SD	<6	35.93 ± 38.76	30.22 ± 23.73	37.90 ± 29.49	0.92

*The normal values are gender-dependent.

### 3.2. mtDNA and nDNA copy numbers in the studied groups

**Figure 1:**
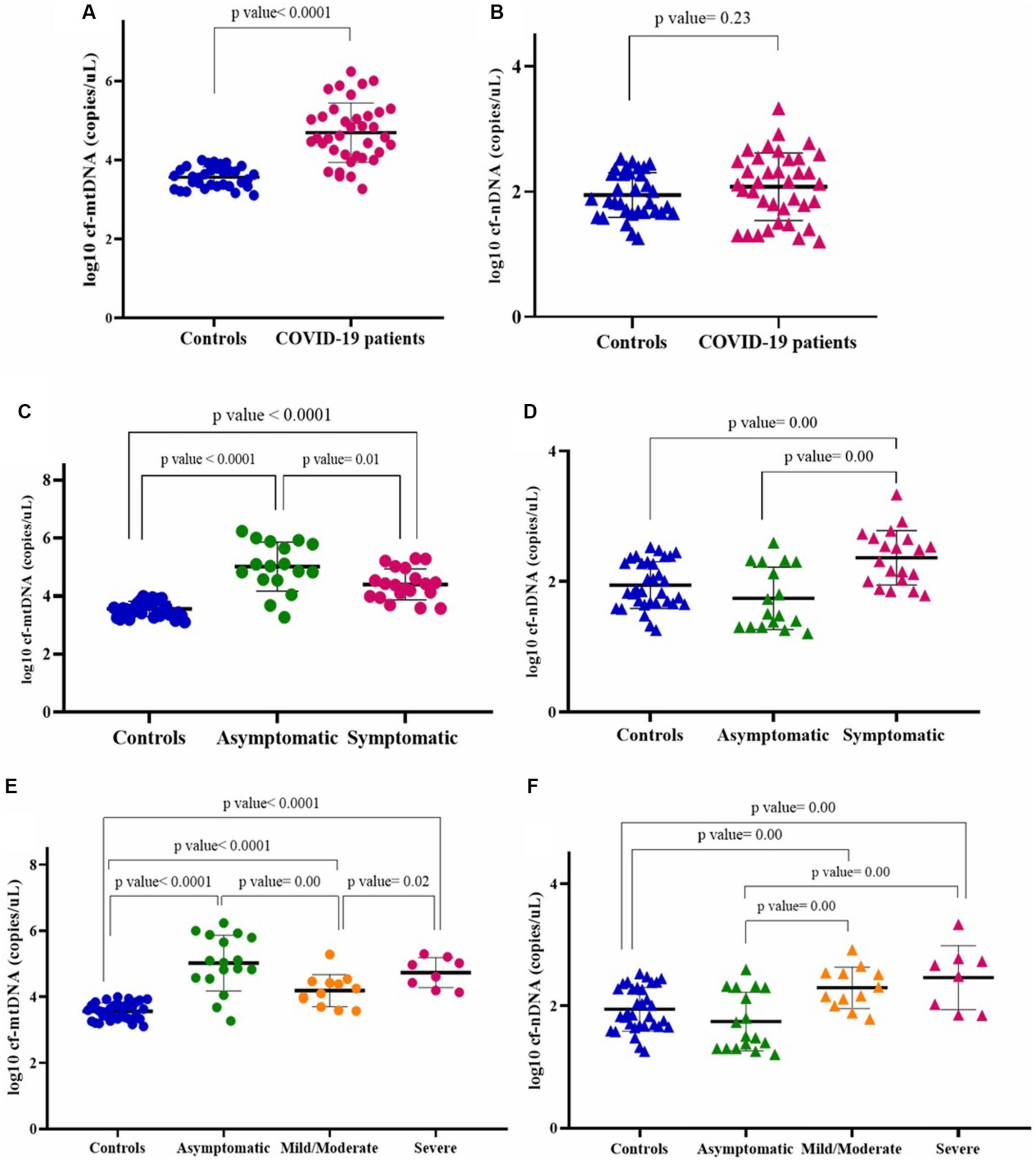
Circulating mtDNA copy number (log10) **(A)**, and circulating nDNA copy number (log10) **(B)** in COVID-19 patients and healthy controls. Circulating mtDNA copy number (log10) **(C)**, and circulating nDNA copy number (log10) **(D)**, in asymptomatic and symptomatic COVID-19 patients. Circulating mtDNA copy number (log10) **(E)**, and circulating nDNA copy number (log10) **(F)**, in asymptomatic, mild/moderate, and severe COVID-19 subgroups.

**COVID-19 patients and healthy controls.** The analysis demonstrated that the COVID-19 group including symptomatic and asymptomatic patients (*n* = 37) had higher plasma levels of *cf*-mtDNA (ND1) compared with those in matched healthy controls (*n* = 33) (log10-transformed data *p* value < 0.05; Figure 1A). The median mtDNA copy numbers in the COVID-19 cases and healthy controls are shown in Table 5.

**Table 5:** The median of copy numbers (non-log10-transformed data) of mtDNA and nDNA in the studied groups.

	Groups			
	COVID-19 patients		Healthy controls	*p* value
mtDNA copies/μL median (Q1,Q3)	37.46 × 10^4^ (13.10 × 10^4^ -145.18 × 10^4^)		3.76 × 10^4^ (2.14 × 10^4^ -5.80 × 10^4^)	**<0.0001**
nDNA copies/μL median (Q1,Q3)	71.00 (46.50–195.50)		133.00 (43.00–333.50)	0.20
	Asymptomatic		Symptomatic	
mtDNA copies/μL median (Q1,Q3)	108.12 × 10^4^ (36.34 × 10^4^ -689.42 × 10^4^)		25.33 × 10^4^ (1.53 × 10^4^ -80.07 × 10^4^)	**0.01**
nDNA copies/μL median (Q1,Q3)	32.00 (20.00–200.50)		254.50 (101.50–459.50)	**0.00**
	Asymptomatic	Mild/moderate	Severe	
mtDNA copies/μL median (Q1,Q3)	108.12 × 10^4^ (36.34 × 10^4^ -689.42 × 10^4^)	15.18 × 10^4^ (5.94 × 10^4^–28.53 × 10^4^)	66.97 × 10^4^ (18.36 × 10^4^–147.96 × 10^4^)	**0.00**
nDNA copies/μL median (Q1,Q3)	32.00 (20.00–200.50)	174.50 (107.50–346.00)	385.00 (79.75–581.50)	**0.00**
	Day 1	Day 3	Day 5	
mtDNA copies/μL median (Q1,Q3)	25.33 × 10^4^ (10.53 × 10^4^–8.07 × 10^4^)	36.34 × 10^4^ (8.93 × 10^4^–167.71 × 10^4^)	36.76 × 10^4^ (16.78 × 10^4^–89.02 × 10^4^)	0.55
nDNA copies/μL median (Q1,Q3)	254.50 (101.50–459.50)	368.00 (187.00–543.00)	221.00 (155.00–381.00)	0.52

Significant *p* values are highlighted.

To obtain the nature of *cf*-DNA, we also determined the circulating nDNA copy number. There was no statistically significant difference in nDNA copy numbers between the COVID-19 cases and controls (log10-transformed data *p* value > 0.05; Figure 1B). The median nDNA copy numbers in these two groups are also demonstrated in Table 5.

**Asymptomatic and symptomatic COVID-19 patients.** We also compared mtDNA copy numbers in asymptomatic (*n* = 17) and symptomatic (*n* = 20) COVID-19 patients. While the mtDNA copy number in both COVID-19 groups was higher than that of controls (*p* values < 0.05), the mtDNA copy number in the asymptomatic COVID-19 was significantly higher than that of the symptomatic group (log10-transformed data *p* value < 0.05; Figure 1C). Circulating nDNA copy numbers were also evaluated in these two groups. The nDNA copy numbers were significantly higher in the symptomatic COVID-19 cases than in the asymptomatic and control groups (log10-transformed data *p* values < 0.05; Figure 1D). The median copy numbers of mtDNA and nDNA in asymptomatic and symptomatic COVID-19 patients are shown in Table 5.

**Asymptomatic, mild/moderate, and severe COVID-19 patients.** As mentioned in the material and method section, patients with COVID-19 were classified into asymptomatic (*n* = 17), mild/moderate (*n* = 12), and severe (*n* = 8) groups based on clinical and laboratory parameters. The results of the comparison of mtDNA copy numbers between these subgroups, which are classified according to disease severity, are shown in Figure 1E.

All these patient subgroups had higher mtDNA copy numbers than the healthy controls (log10-transformed data *p* values < 0.05). The mtDNA copy numbers in the asymptomatic group were higher than the mild/moderate group (log10-transformed data *p* value < 0.05), while there was no significant difference between the asymptomatic and severe cases (log10-transformed data *p* value > 0.05). Also, the *cf*-mtDNA levels in the severe patients were significantly higher than in the mild/moderate subgroup (log10-transformed data *p* value < 0.05).

Circulating nDNA copy numbers were significantly higher in the two symptomatic groups (mild/moderate and severe) than in the asymptomatic group and the controls (log10-transformed data *p* values < 0.05). There was no statistically significant difference between the mild/moderate and severe groups (log10-transformed data *p* value > 0.05) (Figure 1F).

See Table 5 for the median of mtDNA and nDNA copy numbers in COVID-19 subgroups.

**Figure 2:**
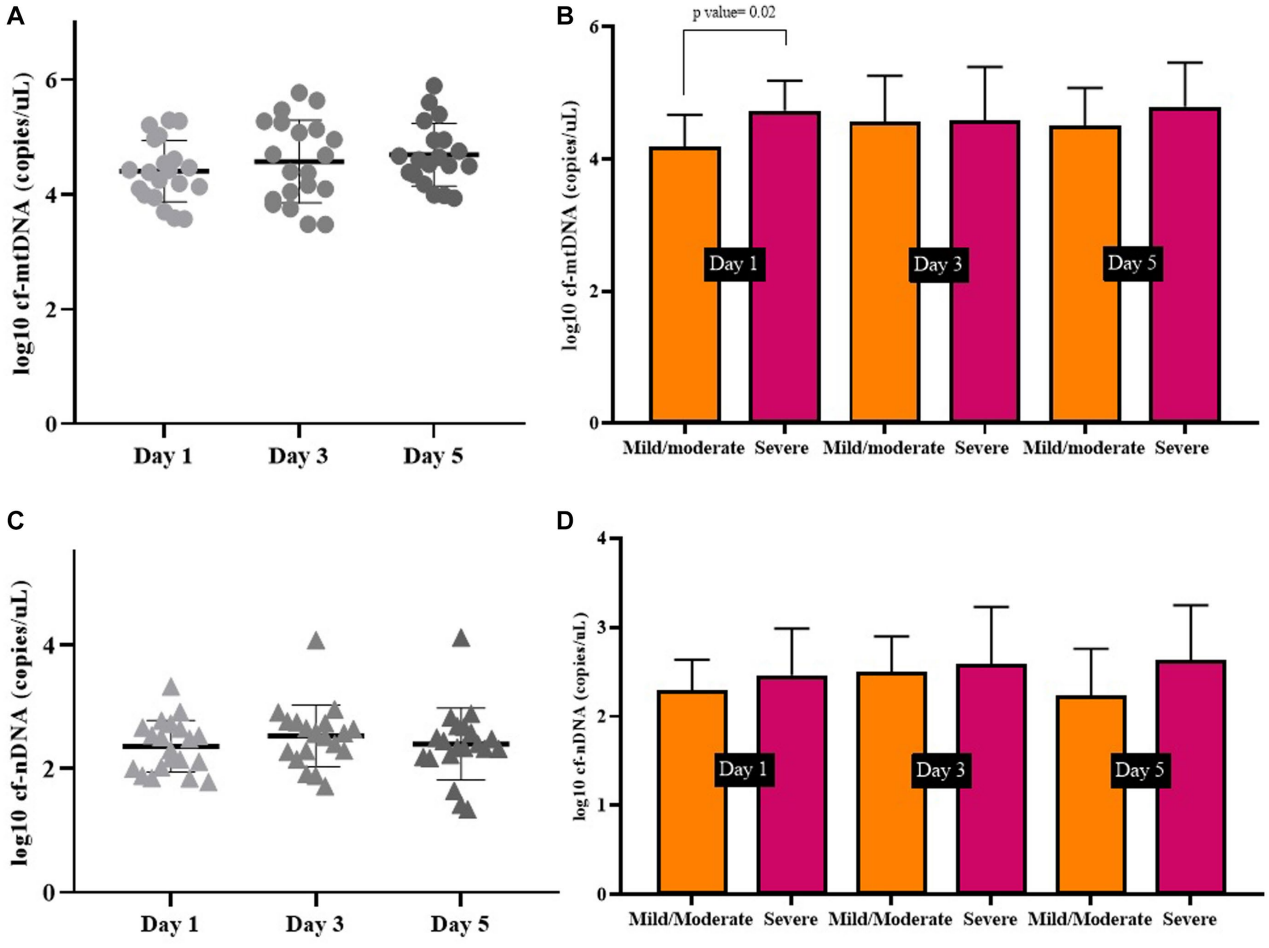
Circulating mtDNA copy number **(A)**, and circulating nDNA copy number **(C)**, on days 1, 3, and 5 in symptomatic COVID-19 patients. Comparison of mtDNA levels **(B)**, and nDNA levels **(D)** on days 1, 3, and 5 in severe and mild/moderate subgroups.

**Days 1, 3, and 5 in symptomatic COVID-19 patients.** We measured the copy number of mtDNA in the plasma of symptomatic COVID-19 patients (*n* = 20) on days 1, 3, and 5, separately. The *cf*-mtDNA levels have been increasing during these 3 days, although this difference was not statistically significant (log10-transformed data *p* value > 0.05; Figure 2A). Similarly, there was no difference between the 3 days in the severe and mild/moderate subgroups (log10-transformed data *p* values > 0.05). We also compared the mtDNA levels in two severe and mild/moderate groups on these 3 days. The mtDNA level of the severe group was significantly higher on the day 1 than the mild/moderate group (log10-transformed data *p* value < 0.05) while there was no significant difference between the days 3 and 5 between these two subgroups (log10-transformed data *p* value > 0.05; Figure 2B).

Also, there was no statistically significant difference between circulating nDNA copy numbers in symptomatic COVID-19 cases on days 1, 3, and 5 (log10-transformed data *p* value > 0.05; Figure 2C). There was no significant difference in nDNA level on the 3 days between severe and mild/moderate subgroups (log10-transformed data *p* value > 0.05; Figure 2D). The median copy numbers of mtDNA and nDNA are given in Table 5.

### 3.3. TFAM mRNA levels in studied groups

**Figure 3:**
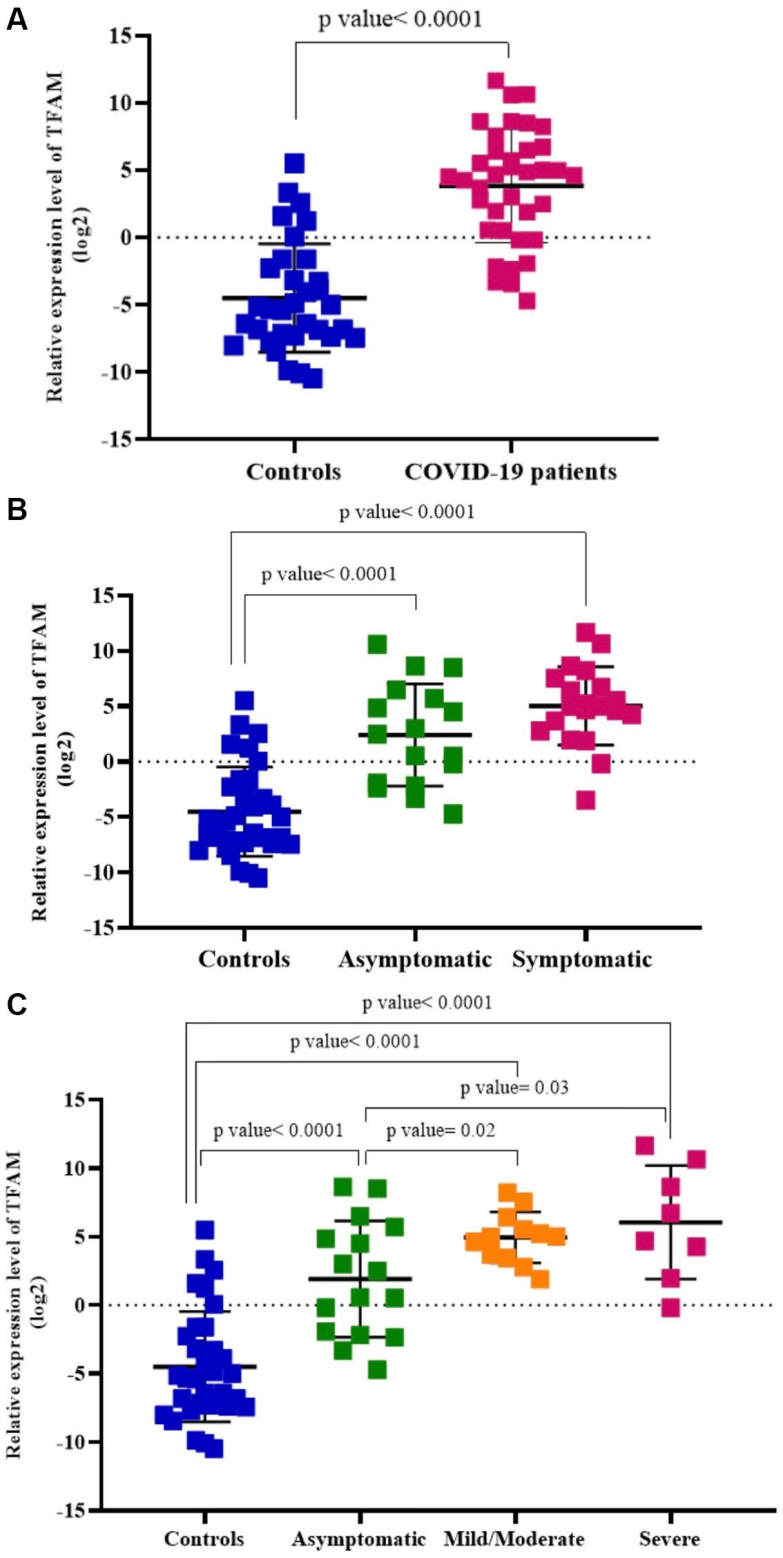
Relative TFAM levels in COVID-19 patients compared to healthy controls **(A)**, asymptomatic and symptomatic COVID-19 patients **(B)**, asymptomatic, mild/moderate, and severe COVID-19 subgroups **(C)**.

**COVID-19 patients and healthy controls.** As shown in Figure 3A, the relative levels of TFAM in the blood of COVID-19 patients (*n* = 37) were significantly higher than those of healthy controls (*n* = 33) (*p* value < 0.05).

**Asymptomatic and symptomatic COVID-19 patients.** TFAM levels were significantly higher in both asymptomatic (*n* = 17) and symptomatic (*n* = 20) COVID-19 patients compared to healthy controls (*n* = 33) (*p* values < 0.05). There was no significant difference between asymptomatic patients and those with symptoms (*p* value > 0.05; Figure 3B).

**Asymptomatic, mild/moderate, and severe COVID-19 patients.** TFAM levels in the asymptomatic group (*n* = 17) were significantly lower than the mild/moderate (*n* = 12) and severe (*n* = 8) groups (*p* values < 0.05). There was no significant difference between the mild/moderate patients and the severe group (*p* value > 0.05) (Figure 3C).

**Figure 4:**
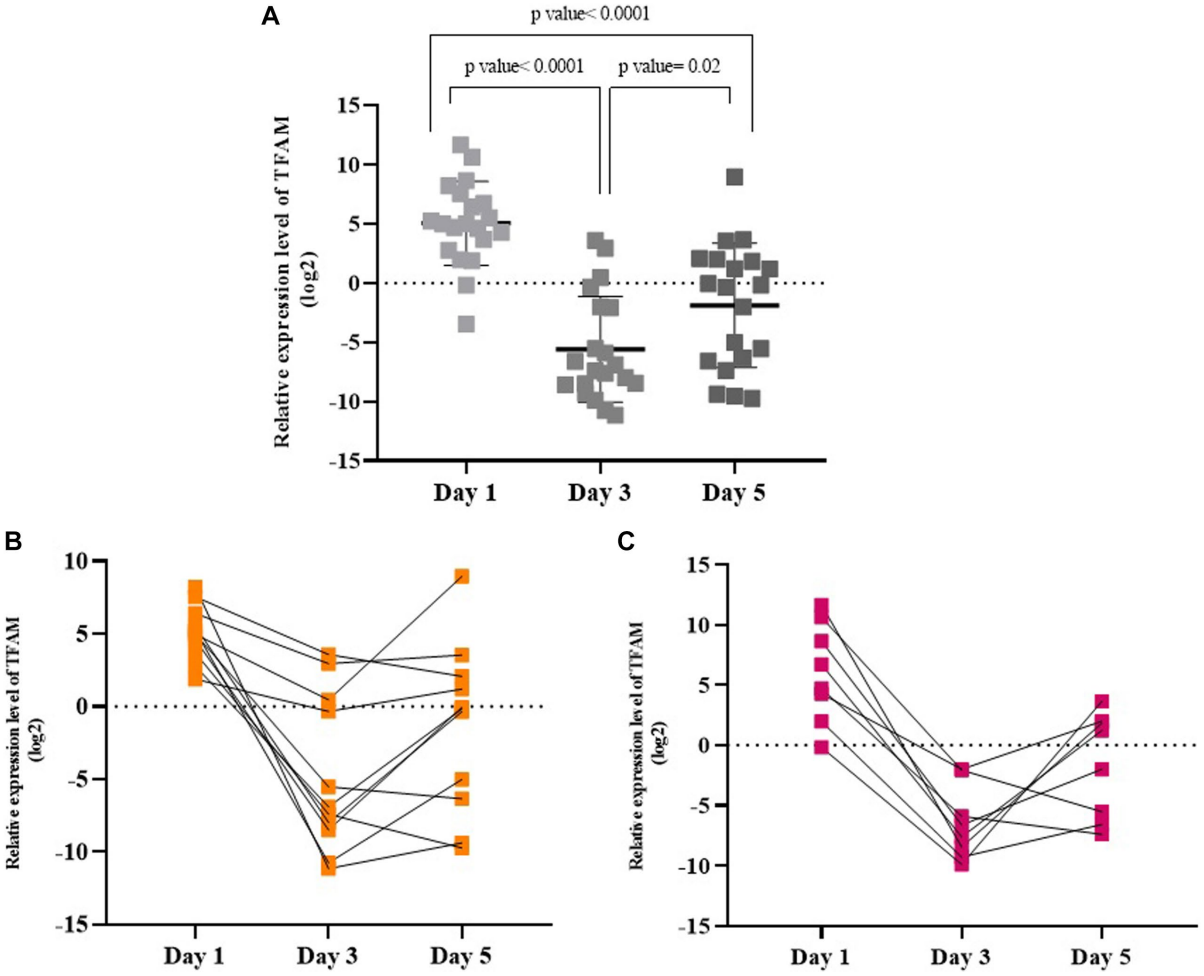
Relative TFAM levels on days 1, 3, and 5 in symptomatic COVID-19 patients **(A)**, in mild/moderate **(B)**, and in severe **(C)** subgroups.

**Days 1, 3, and 5 in symptomatic COVID-19 patients.** The TFAM levels were significantly higher on day 1 than on days 3 and 5 (*p* values < 0.05). Also, as shown in Figure 4A, the TFAM levels on day 3 were at the lowest level compared to day 1 and day 5 (*p* values < 0.05). TFAM levels in 3 days were evaluated in mild/moderate and severe subgroups. The TFAM levels in the mild/moderate group on day 1 was significantly higher than days 3 and 5 (*p* values < 0.05; Figure 4B), while there was no significant difference between days 3 and 5 (*p* value > 0.05). Similarly, the TFAM level in the severe cases on day 1 was significantly higher than days 3 and 5 (*p* values < 0.05; Figure 4C), while there was no significant difference between days 3 and 5 (*p* value > 0.05). However, there was no significant difference in the TFAM levels between the severe and mild/moderate groups in any of the 3 days (*p* value > 0.05).

### 3.4. Correlations

**Correlation between *cf*-mtDNA, *cf*-nDNA, TFAM levels, and clinical parameters.** Spearman correlation was performed to investigate the possible relationship between important clinical parameters in COVID-19 with mtDNA, nDNA copy numbers, and TFAM levels. The results are presented in Table 6.

**Table 6:** Correlation between *cf*-mtDNA, *cf*-nDNA, TFAM levels, and significant clinical parameters.

Parameters	mtDNA copy number (log10)		nDNA copy number (log10)		TFAM mRNA level (log2)	
	*r*	*p* value	*r*	*p* value	*r*	*p* value
Symptomatic COVID-19						
Age (year)	0.42	0.06	0.43	0.06	−0.01	0.93
D-Dimer (ng/mL)	0.37	0.23	−0.15	0.63	0.36	0.23
CRP (mg/L)	−0.21	0.39	0.35	0.14	−0.04	0.85
LDH (IU/L)	0.13	0.65	0.40	0.16	−0.28	0.35
ESR (mm/h)	−0.04	0.86	−0.01	0.94	0.16	0.50
Neutrophil (%)	0.24	0.52	0.11	0.76	0.19	0.61
Mild/moderate subgroup						
Age (year)	0.37	0.23	0.35	0.24	0.14	0.66
D-Dimer (ng/mL)	0.03	0.96	0.46	0.30	0.00	0.90
CRP (mg/L)	−0.39	0.25	0.23	0.51	0.07	0.84
LDH (IU/L)	0.07	0.88	0.61	0.11	−0.26	0.53
ESR (mm/h)	−0.19	0.53	−0.12	0.71	0.02	0.95
Neutrophil (%)	0.40	0.75	0.80	0.33	−0.20	0.91
Severe subgroup						
Age (year)	−0.14	0.75	0.38	0.35	−0.45	0.26
D-Dimer (ng/mL)	0.30	0.68	−0.80	0.13	0.30	0.68
CRP (mg/L)	0.04	0.93	0.64	0.09	−0.40	0.32
LDH (IU/L)	0.50	0.45	0.10	0.95	−0.80	0.13
ESR (mm/h)	0.28	0.55	0.07	0.90	0.17	0.71
Neutrophil (%)	0.20	0.78	−0.50	0.45	0.20	0.78

**Correlation between TFAM mRNA levels and mtDNA copy number.** Also, to investigate the relationship between the copy numbers of mtDNA and the mitochondrial transcription factor (TFAM), the correlation analysis was performed on all studied groups. There was no significant relationship between TFAM mRNA levels and *cf*-mtDNA copy numbers in any of the groups (*p* values > 0.05).

### 3.5. Diagnostic value of *cf*-mtDNA, *cf*-nDNA, and TFAM mRNA levels in studied groups

**Figure 5:**
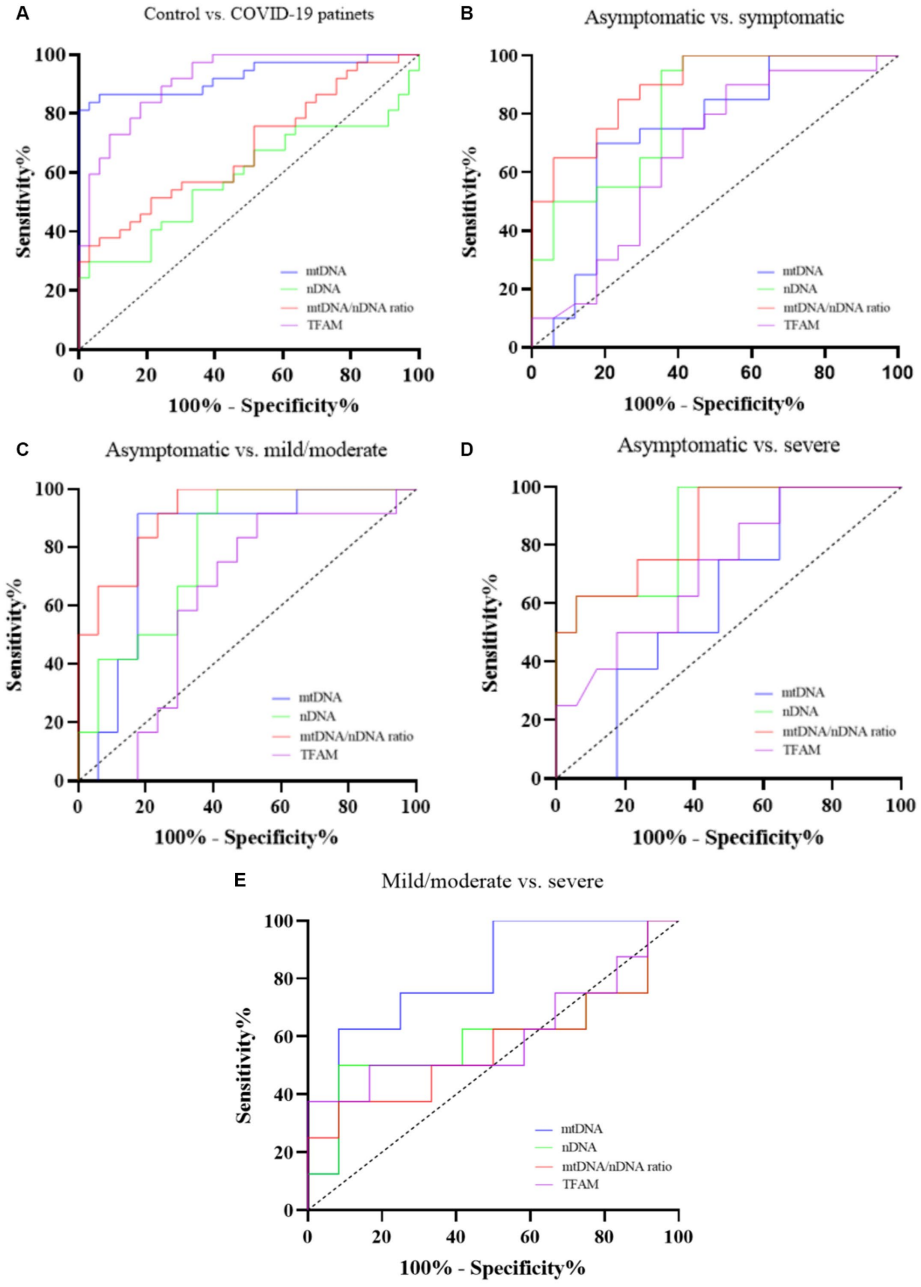
ROC curve of *cf*-mtDNA, *cf*-nDNA, mtDNA/nDNA ratio, and TFAM between Controls and COVID-19 patients **(A)**, Asymptomatic and symptomatic patients **(B)**, Asymptomatic and mild/moderate patients **(C)**, Asymptomatic and severe patients **(D)**, and Mild/moderate and severe patients **(E)**.

A receiver operating characteristic curve (ROC) analysis was performed to evaluate the diagnostic values of mtDNA, nDNA copy number, and TFAM levels. We also examined the diagnostic value of the mtDNA/nDNA ratio. Details of the ROC curve analysis are presented in Table 7 and Figure 5.

**Table 7:** Diagnostic value of *cf*-mtDNA, *cf*-nDNA, mtDNA/nDNA ratio, and TFAM mRNA levels.

Marker	AUC ± SD	*p* value	*95%* CI		Cutoff value	Sensitivity (%)	Specificity (%)
			Lower	Upper			
COVID-19 patients and controls							
mtDNA copy number (log10)	0.92 ± 0.03	**<0.0001**	0.86	0.99	>3.65	91.89	60.61
nDNA copy number (log10)	0.58 ± 0.06	0.21	0.44	0.72	>2.10	54.05	66.67
mtDNA/nDNA ratio	0.68 ± 0.06	**0.00**	0.55	0.80	>2.18	51.35	78.79
TFAM levels	0.91 ± 0.03	**<0.0001**	0.85	0.97	>0.21	89.19	75.76
Asymptomatic and symptomatic							
mtDNA copy number (log10)	0.73 ± 0.08	**0.01**	0.56	0.91	<4.54	70.00	82.35
nDNA copy number (log10)	0.82 ± 0.06	**0.00**	0.68	0.96	>1.82	95.00	64.71
mtDNA/nDNA ratio	0.89 ± 0.05	**<0.0001**	0.79	0.99	<1.93	65.00	94.12
TFAM levels	0.66 ± 0.09	0.08	0.48	0.84	>10.45	75.00	58.82
Asymptomatic and mild/moderate							
mtDNA copy number (log10)	0.81 ± 0.08	**0.00**	0.65	0.98	<4.54	91.67	82.35
nDNA copy number (log10)	0.79 ± 0.08	**0.00**	0.63	0.95	>1.75	100.00	58.82
mtDNA/nDNA ratio	0.91 ± 0.04	**0.00**	0.81	1.00	<2.41	100.00	70.59
TFAM levels	0.62 ± 0.10	0.24	0.41	0.83	>6.24	83.33	52.94
Asymptomatic and severe							
mtDNA copy number (log10)	0.61 ± 0.11	0.35	0.39	0.83	<5.02	75.00	52.94
nDNA copy number (log10)	0.86 ± 0.07	**0.00**	0.70	1.00	>1.82	100.00	64.71
mtDNA/nDNA ratio	0.86 ± 0.07	**0.00**	0.70	1.00	<1.93	62.50	94.12
TFAM levels	0.72 ± 0.10	0.07	0.51	0.93	>13.65	75.00	58.82
Mild/moderate and severe							
mtDNA copy number (log10)	0.80 ± 0.10	**0.02**	0.60	1.00	>4.11	100.00	50.00
nDNA copy number (log10)	0.59 ± 0.14	0.48	0.30	0.88	>2.39	62.50	58.33
mtDNA/nDNA ratio	0.56 ± 0.14	0.64	0.27	0.84	>1.90	50.00	66.67
TFAM levels	0.60 ± 0.14	0.44	0.32	0.88	>25.29	62.50	41.67

Significant *p* values are highlighted.

## 4. Discussion

Clinical outcomes of SARS-CoV-2 infection may vary from asymptomatic to severe forms with complications (Kouhpayeh, 2022). Severe clinical manifestations of COVID-19 have often been associated with a hyper-inflammatory state and impaired antiviral response (Neufeldt et al., 2022). Strong evidence suggests that mitochondria have a prominent role in regulating the immune responses to pathogens, the pro-inflammatory response, and cell death (West et al., 2011). Many viruses modulate the mitochondria to evade the mitochondrial-mediated antiviral response (Tiku et al., 2020; Newman and Shadel, 2023). Mitochondrial structure alteration and dysfunction have been reported in SARS-CoV-2 infected airway epithelial cells in addition to endothelial cells, monocytes, and T lymphocytes (Gibellini et al., 2020; Domizio et al., 2022; Mo et al., 2022; Yang et al., 2022). A growing number of studies have suggested COVID-19-induced mitochondrial damage as a pathological factor in SARS-CoV-2 infection (Gibellini et al., 2020; Tian et al., 2021; Domizio et al., 2022). On the other hand, mitochondrial damage-associated molecular patterns (mtDAMPs), such as mtDNA are potent immune activators that are mainly released into the extracellular space due to mitochondrial structural and functional alterations (Bock and Tait, 2020). The released mtDNA can activate various pro-inflammatory pathways such as TLR9, inflammasome, and cGAS-STING. Previous studies have shown that some viral infections could lead to mitochondrial stress and the release of mtDNA into the extracellular space (Riley and Tait, 2020). A recent study demonstrated that SARS-CoV-2 infection of endothelial cells caused mitochondrial dysfunction, increased mtDNA release, and activation of TLR9 signaling (Costa et al., 2022). Also, high levels of *cf*-mtDNA have been reported in COVID-19 cases and have been associated with an increased risk of ICU admission and mortality (Scozzi et al., 2021; Valdés-Aguayo et al., 2021). However, there were few studies on *cf*-mtDNA levels in SARS-CoV-2 infection, which have mainly focused on critically ill COVID-19 patients. The strength of our study is that we evaluated *cf*-mtDNA levels in both asymptomatic and symptomatic COVID-19 patients. In addition, we also measured *cf*-nuclear DNA and TFAM mRNA levels.

According to our results, *cf*-mtDNA levels in COVID-19 patients (including asymptomatic and symptomatic cases) were higher compared with healthy controls. Also, there was a statistically significant difference between the asymptomatic and symptomatic COVID-19 groups. Interestingly, *cf*-mtDNA copy numbers in the asymptomatic group had higher levels compared to the symptomatic cases. We also compared *cf*-mtDNA copy numbers in three subgroups of COVID-19 including asymptomatic, mild/moderate, and severe. The results showed significantly higher levels of *cf*-mtDNA in all three subgroups compared to the healthy controls. In addition, the asymptomatic group had higher *cf*-mtDNA levels than the mild/moderate group, and the *cf*-mtDNA copy numbers were significantly higher in the severe group than in the mild/moderate group. The *cf*-mtDNA levels have been increasing at 3 days follow-up after enrollment, although this difference was not statistically significant. The mtDNA levels on the day 1 in the severe group was significantly higher than those of the mild/moderate.

A previous study measured circulating plasma mtDNA levels (using the mt-cytb gene) in 97 hospitalized COVID-19 patients. They reported that plasma mtDNA levels were significantly increased in those patients who died of COVID-19 or who required ICU admission or intubation (Scozzi et al., 2021). Although this study did not have a control group and did not classify COVID-19 patients, consistent with our results, it reported higher levels of *cf*-mtDNA in severe cases than in other symptomatic patients. Another study by Edinger et al. (2022) showed higher plasma mtDNA levels (using the ND1 gene) in 29 critically ill COVID-19 patients treated in the ICU (363 copies/μL) compared to 29 healthy controls (65 copies/μL). The assessment of plasma mtDNA concentrations (using the ND1 and the mt-cytB) in 20 critically ill COVID-19 patients by droplet digital PCR (ddPCR) also provided similar results. The mtDNA levels were significantly increased in COVID-19 patients with moderate/severe acute respiratory distress syndrome (ARDS) compared to patients without/mild ARDS. Also, the highest concentrations of mtDNA were observed over time (on days 1, 3, 5, 7, 9, and 11 after enrollment) in more severe COVID-19 cases (Hepokoski et al., 2022).

The higher levels of mtDNA in COVID-19 patients compared to controls observed in our results could be attributed to the mitochondrial damage caused by SARS-CoV-2 infection. A recent study has shown that the SARS-CoV-2 ORF3a induces mitochondrial damage and mitochondrial reactive oxygen species (mtROS) generation (Tian et al., 2021). Faizan et al. also demonstrated that NSP4 and ORF9b of SARS-CoV-2 could induce mitochondrial structural changes, formation of macropores in the outer membrane, and the release of inner membrane vesicles loaded with mitochondrial DNA in primary human airway epithelial cells. They also reported that the mtDNA copy number in the plasma of COVID-19 patients was higher than that of the convalescent and the control groups (Faizan et al., 2022). A large number of mtDNA copies appear to compensate for damage, so a high level of mitochondrial DNA could be a marker for mitochondrial function and oxidative stres (Riou et al., 2020). Therefore, the high mtDNA levels may reflect mitochondrial damage induced by inflammation and oxidative stress in SARS-CoV-2 infection. Since the main hallmark of the severity of COVID-19 is the hyper-inflammatory response and oxidative stress (Vollbracht and Kraft, 2022), our finding of higher mtDNA levels in severe COVID-19 cases compared to mild/moderate patients could be justified.

According to our other findings about the difference in *cf*-mtDNA levels in asymptomatic and symptomatic COVID-19 patients, it is worth mentioning the molecular characteristics of these two groups. To date, there is no clear answer to the question of what determines asymptomatic or symptomatic manifestations after SARS-CoV-2 infection (Wang et al., 2022). Also, limited data is available about the infectivity of asymptomatic COVID-19 cases (Sayampanathan et al., 2021). The pattern of viral shedding in asymptomatic COVID-19 patients suggests that these cases are infectious (Lee et al., 2020). Most researches showed that asymptomatic cases shed infectious SARS-CoV-2 faster than symptomatic patients. Also, viral clearance is more rapid in asymptomatic COVID-19 patients, resulting in a shorter duration of infectiousness (Nogrady, 2020; Cevik et al., 2021). Interestingly, persistent shedding of SARS-CoV-2 RNA has been reported in asymptomatic cases (Li et al., 2020). Although a higher viral load is associated with a longer duration of viral shedding, asymptomatic COVID-19 patients in most cases shed the virus faster than symptomatic patients, even if their viral loads are similar (Xiao et al., 2021). This may be related to various factors including host factors, such as age, co-morbidities, and immune response (Fontana et al., 2021). A previous study suggested that the lower SARS-CoV-2 RNA load and shorter duration of viral shedding in asymptomatic COVID-19 cases are likely due to their stronger antiviral immunity. They also postulated a greater role for innate and adaptive cellular immunity than for humoral immunity because IgM or IgG neutralizing antibody declines faster in asymptomatic COVID-19 than in symptomatic cases (Chen et al., 2021). However, the key question about asymptomatic infection is whether it is a mild infection or an adequate immune control to suppress symptoms. A comparative analysis of immune phenotype using single-cell RNA sequencing in asymptomatic, mild, or severe COVID-19 supports the idea that innate immunity is altered in asymptomatic compared to symptomatic infection. Expression of IFN-I-related genes in PBMCs of asymptomatic cases was lower than that of severe patients. This may indicate that a fast and effective IFN-I response is involved in asymptomatic infection, whereas in symptomatic individuals a longer and more pronounced response controls the virus less effectively. They suggested that the early innate immune response and IFN-I may have a role in the asymptomatic phenotype of COVID-19 (Zhao et al., 2020). Another study analyzing SARS-CoV-2-specific T cells in asymptomatic and symptomatic COVID-19 patients, showed that the levels of IFNγ, interleukins-2, 6, and TNF were higher in the asymptomatic cases than in the symptomatic patients. It can therefore be argued that, even in the absence of symptomatic infection, SARS-CoV-2 induces a high frequency of effector CD4^+^ T cells that produce high levels of pro-inflammatory cytokines (Boyton and Altmann, 2021; Le Bert et al., 2021). A previous study showed that the plasma cytokine levels such as IL-6, IL-1β, IL-10, IL-21, and TNF-α were significantly higher in asymptomatic group compared to the controls, indicating an increased inflammation in asymptomatic COVID-19 patients. Upregulation of ISGs and humoral immunity genes was demonstrated in asymptomatic COVID-19 cases. Additionally, responses to type I interferon in asymptomatic COVID-19, compared to mild and severe cases, were significantly modulated by dysregulation of some ISGs associated with progressive disease (Masood et al., 2021).

The dual role of IFN-I in both immunosuppression and inflammation may be involved in the pathogenesis of COVID-19. While early activation of IFN-I in primary acute infection leads to viral suppression by antiviral innate immunity, delayed but high levels of IFN-I lead to viral persistence, uncontrolled inflammation, and severe disease (Marcinkiewicz et al., 2021; Nazerian et al., 2022; Darif et al., 2023). The ability of the released mtDNA to induce the IFN production through the activation of the cGAS-STING cascade (Riley and Tait, 2020) may justify the high levels of mtDNA in the asymptomatic group in our study. As interferon signaling might be protective in the early stages of infection but pathological in the late stages (Metcalf et al., 2020; Mahmoodpoor et al., 2022), the role of *cf*-mtDNA and the timing of immune responses in COVID-19 outcome should be further studied.

Although the main aim of our study was not to identify the mechanisms that cause the release of mtDNA, we also evaluated the *cf*-nDNA copy numbers to obtain the nature of *cf*-DNA. According to our results, there was no significant difference in the nDNA copy numbers between COVID-19 patients and controls. *cf*-nDNA copy numbers were also significantly higher in the symptomatic COVID-19 group than in the asymptomatic and control groups. The nDNA levels in the two symptomatic groups (mild/moderate and severe) were significantly higher than the asymptomatic group and the control group. There was no significant difference between the mild/moderate and severe groups. Furthermore, there was no significant difference between nDNA copy numbers in symptomatic COVID-19 cases on days 1, 3, and 5.

Since *cf*-DNA mainly originates from apoptotic and necrotic cells, high *cf*-DNA levels may reflect tissue damage and inflammation (Gravina et al., 2016). Nuclear DNA levels increase in pathological damage to cells, while *cf*-mtDNA originates from damaged mitochondria and reflects mitochondrial condition (Filograna et al., 2021). To date, little is known about the molecular mechanisms that lead to the release of mtDNA into the extracellular environment. Release of mtDNA during apoptosis and pyroptosis, but independent of cell death pathways, has also been reported. Mitochondrial stress, mtDNA stress, some immune signals and viral infections are among the factors inducing mtDNA release (Heilig et al., 2023). Studies have suggested that mtDNA can be released by a passive or accidental process such as cell necrosis or apoptosis, or by an active or regulated mechanism including NETosis. Although both pathways are pathophysiologically related, the relative contribution of active versus passive release remains unclear. Neutrophils are one of main sources of extracellular mtDNA. Recent studies have demonstrated the important role of neutrophil extracellular traps (NETs) in the pathogenesis of COVID-19 (De Gaetano et al., 2021; Al-Kuraishy et al., 2022). However, the source and mechanism of mtDNA release in SARS-CoV-2 infection are still unclear. A recent study evaluated the specific parameters of NETs on 91 hospitalized patients with COVID-19 including *cf*-DNA, MPO-DNA, and NE-DNA complexes and citrullinated Histone 3 (citH3). They suggested that sources of *cf*-DNA in hospitalized COVID-19 patients were more associated with tissue injury than NETs (De Gopegui et al., 2022). Also, another study aimed to examine whether mtDNA release is associated with mitochondrial damage caused by SARS-CoV-2 infection. They measured the plasma levels of pro-apoptotic Cytochrome c (Cyt c), which indicates mitochondrial damage. Consistent with mtDNA release findings, plasma Cyt c levels were elevated in COVID-19 cases. These findings demonstrated that *cf*-mtDNA is a strong indicator of mitochondrial damage in COVID-19 and could be utilized as a potential biomarker together with other pro-inflammatory markers to indicate the disease severity (Faizan et al., 2022). SARS-CoV-2 can directly or indirectly cause the death of airway epithelial cells. In direct virus-induced cell death, viral proteins lead to the activation of pro-apoptotic signaling by hijacking host cell anti-apoptotic proteins. On the other hand, the activation of DAMPs and pro-inflammatory cytokines mediate the indirect cell death (Faizan et al., 2022). Our findings also showed that plasma nDNA levels are higher in severe and mild/moderate groups compared to the asymptomatic group, it could be concluded that in addition to the selective release of mtDNA, cell death may also have occurred in severe COVID-19 cases (Yuzefovych et al., 2019). Persistent expression of inflammatory cytokines in critically ill COVID-19 patients may lead to an increased influx of neutrophils, which are the source of tissue damage (Masood et al., 2021). Oxidation of mtDNA following mitochondrial dysfunction and subsequent ROS generation can activate inflammasomes during SARS-CoV-2 infection. Activation of inflammasomes has promoted the pyroptosis of infected monocytes/macrophages (Junqueira et al., 2021; Nazerian et al., 2022). A recent study identified T cell apoptosis as the cause of SARS-CoV-2 infection-mediated lymphopenia in severe COVID-19 disease (André et al., 2022). Another study has suggested the association of plasma mtDNA with apoptosis and necrosis of endothelial cells (Costa et al., 2022). In addition, cytokine storm and oxidative stress lead to excessive ROS generation, which induces mitochondrial damage, apoptosis, and mtDNA leakage (Costa et al., 2022). Cytokines such as IL-6 and TNF-α, found in the serum of severe COVID-19 patients, cause decreased ATP production and abnormal generation of mtROS. This causes alterations in mitochondrial membrane permeability, mtDNA releasing and mitochondrial dynamics, and ultimately could lead to cell death (Valdés-Aguayo et al., 2021). Our findings showed a higher level of mtDNA and a lower level of nDNA in the asymptomatic compared to the symptomatic COVID-19 patients. This may be related to the different mechanism of mtDNA release in these groups. However, further research is required to deeply understand the underlying molecular mechanism(s).

Since molecular and genetic models have shown a direct relationship between mitochondrial transcription factor A (TFAM) and mtDNA levels, in the following, we investigated TFAM mRNA levels in the buffy coat of the studied groups. TFAM levels in the COVID-19 patients increased significantly compared to the control group. Also, the levels of TFAM in both symptomatic and asymptomatic groups increased compared to controls, but there was no significant difference between these two COVID-19 groups. Comparison of the relative expression of TFAM in the COVID-19 subgroups showed higher levels in severe and mild/moderate subgroups compared to asymptomatic cases. There were no significant differences between the severe and mild/moderate subgroups. Likewise, TFAM levels in symptomatic patients were significantly higher on day 1 than on days 3 and 5. Similarly, the TFAM levels in the mild/moderate and severe groups on day 1 was significantly higher than days 3 and 5, whereas there was no significant difference between days 3 and 5.

Mitochondrial transcription factor A (TFAM) is a mitochondrial DNA-binding protein encoded in the nuclear genome. The role of TFAM is to determine mitochondrial genome abundance by regulating mtDNA replication, transcription, and packaging. Recent studies have also suggested a central role of TFAM in the inflammatory response induced by mtDNA stress. Mitochondrial dysfunction could lead to upregulation in the transcription of nuclear-encoded mitochondrial genes as a compensatory strategy. This process is known as mitochondrial biogenesis, which is an increase in mitochondrial content that is often mediated by changes in nuclear transcription. However, the increase in mtDNA copy number can be separated from mitochondrial biogenesis, which means that mtDNA copy number can increase without increasing the number of mitochondria. In most cases, upregulation of TFAM transcription is associated with increased mtDNA (Kang et al., 2018; Filograna et al., 2021). Conversely, a 2019 study suggested that mtDNA content does not always correlate with TFAM expression and that the effects of changes in TFAM expression may be cell-type specific. Accordingly, TFAM expression alteration should be cautiously attributed to mitochondrial biogenesis (Kozhukhar and Alexeyev, 2019). Interestingly, *in vitro* and *in vivo* evidence showed that titration of TFAM overexpression increased mtDNA levels at low TFAM levels, but decreased at higher levels. This suggests that overcompaction of the mitochondrial genome inhibits replication (Farge et al., 2014; Bonekamp et al., 2021). In addition, mtDNA degradation caused by the depletion of TFAM protein leads to the cytoplasmic release of mtDNA and thus triggers antiviral responses via the cGAS-STING pathway (Sato et al., 2021). In our study, there was also no significant correlation between TFAM levels in the buffy coat and mtDNA copy numbers in plasma. This finding could be due to the presence of sources other than white blood cells releasing mtDNA in plasma.

Delay or failure to activate mitochondrial biogenesis early in the disease may increase susceptibility to mitochondrial oxidative damage. This impairment may affect the ability to recover normal function (Carré et al., 2010). A previous study conducted on septic patients suggested that measurement of mitochondrial biogenesis gene expression markers including TFAM in PBMCs follows a distinct timeline of activation and may have potential to predict recovery. They showed that early activation of mitochondrial biogenesis in PBMCs is associated with clinical improvement and discharged from the ICU (Kraft et al., 2019).

Consistent with other studies on sepsis, we found an upregulation of TFAM mRNA in symptomatic COVID-19 cases compared to asymptomatic and healthy controls. However, this early increase in TFAM levels may not be a reflection of mitochondrial recovery. As a previous study showed, despite the increase in extramitochondrial TFAM, the frequency of intramitochondrial TFAM decreases in PBMCs from patients with sepsis. This result may help explain the paradox of lacking bioenergetic recovery despite increased TFAM expression (Rahmel et al., 2020).

To evaluate the diagnostic value, we used ROC curve analysis. Many studies have proposed *cf*-mtDNA as a biomarker for diseases including viral infections. *cf*-mtDNA is also a candidate biomarker for pathogen-induced cell death and disease severity (Gambardella et al., 2019). Previous studies conducted in critically ill COVID-19 patients have shown that *cf*-mtDNA levels are a potential biomarker for diagnosing disease severity. Edinger et al. (2022) showed excellent predictive performance of in-hospital COVID-19 mortality for ND1 mtDNA levels (AUC: 0.90, sensitivity: 86% and specificity: 100%). Another study suggested cytB mtDNA levels as an early indicator of a higher risk of COVID-19 mortality (AUC: 0.68) and ICU admission (AUC: 0.75) and intubation (AUC: 0.86) (Scozzi et al., 2021). Also, in a recent study, the AUC comparing PBMC mt-DNA concentration of healthy controls with mild to moderate COVID-19 patients (non-severe cases) was 0.76 (sensitivity: 84% and specificity: 62%). However, mtDNA levels were not statistically significant when comparing severe COVID-19 cases who died or recovered (AUC: 0.32) (Valdés-Aguayo et al., 2021). These findings are consistent with our results.

Increasing studies have addressed the importance of mitochondrial function and mtDNA levels in SARS-CoV-2 infection. Mitochondrial dysfunction has been suggested as one of the underlying mechanisms of long-COVID (Chen et al., 2023). Also, the association of mitochondria with conditions that increase the risk of COVID-19 mortality, such as aging and metabolic disorders, has been proposed (Ganji and Reddy, 2021; Srinivasan et al., 2021; Yu et al., 2022). Recently, attention has been paid to the effect of mitochondrial DNA levels on the immune response after COVID-19 vaccination. Peripheral mtDNA copy number is positively correlated with higher IgG (anti-spike) titers and cell-mediated immune responses (Ikezaki et al., 2023). These results highlight the role of mitochondria in SARS-CoV-2 infection, which requires further investigation.

In conclusion, our study showed higher levels of *cf*-mtDNA in asymptomatic COVID-19 patients than in symptomatic cases. Therefore, mitochondrial DAMPs and related events are important in the outcome of the disease. More studies aimed at unraveling the underlying mechanisms may provide a better understanding of the COVID-19 pathophysiology and effective diagnostic and therapeutic strategies.

### 4.1. Study limitation

One of the limitations of our study was the absence of a group that had COVID-19-like symptoms, but whose RT-qPCR and CT scan were negative for SARS-CoV-2 infection.

## Data availability statement

The original contributions presented in the study are included in the article/Supplementary material, further inquiries can be directed to the corresponding authors.

## Ethics statement

The studies involving humans were approved by Research Institute for Gastroenterology and Liver Disease (RIGLD) in Tehran, Iran (#IR.SBMU.RIGLD.1399.008). The studies were conducted in accordance with the local legislation and institutional requirements. The participants provided their written informed consent to participate in this study.

## Author contributions

SHSH: Methodology, Investigation, Formal Analysis, Writing – original draft. SRM: Supervision, Conceptualization, Validation, Data curation, Formal Analysis, Writing – review & editing. SMH: Supervision, Conceptualization, Project administration, Data curation, Formal Analysis, Writing – review & editing. MRZ: Resources, Funding acquisition, Writing – review & editing.
